# Meeting Report: Consensus Statement—Parkinson’s Disease and the Environment: Collaborative on Health and the Environment and Parkinson’s Action Network (CHE PAN) Conference 26–28 June 2007

**DOI:** 10.1289/ehp.11702

**Published:** 2008-08-26

**Authors:** Jeff Bronstein, Paul Carvey, Honglei Chen, Deborah Cory-Slechta, Donato DiMonte, John Duda, Paul English, Samuel Goldman, Stephen Grate, Johnni Hansen, Jane Hoppin, Sarah Jewell, Freya Kamel, Walter Koroshetz, James W. Langston, Giancarlo Logroscino, Lorene Nelson, Bernard Ravina, Walter Rocca, George W. Ross, Ted Schettler, Michael Schwarzschild, Bill Scott, Richard Seegal, Andrew Singleton, Kyle Steenland, Caroline M. Tanner, Stephen Van Den Eeden, Marc Weisskopf

**Affiliations:** 1 UCLA School of Medicine, Los Angeles, California, USA; 2 Rush University Medical Center, Chicago, Illinois, USA; 3 National Institute of Environmental Health Sciences, National Institutes of Health, Department of Health and Human Services, Research Triangle Park, North Carolina, USA; 4 University of Rochester School of Medicine and Dentistry, Rochester, New York, USA; 5 The Parkinson’s Institute and Clinical Center, Sunnyvale, California, USA; 6 Parkinson’s Disease Research, Education, and Clinical Center, Philadelphia, Pennsylvania, USA; 7 California Department of Health Services, Oakland, California, USA; 8 U.S. Army Medical Research and Material Command, Fort Detrick, Maryland, USA; 9 Institute of Cancer Epidemiology, Danish Cancer Society, Copenhagen, Denmark; 10 National Institute of Neurological Disorders and Stroke, National Institutes of Health, Department of Health and Human Services, Bethesda, Maryland, USA; 11 University of Bari, Bari, Italy; 12 Stanford University School of Medicine, Stanford, California, USA; 13 University of Rochester School of Medicine, Rochester, New York, USA; 14 Mayo Clinic, Rochester, Minnesota, USA; 15 Pacific Health Research Institute, Honolulu, Hawaii, USA; 16 Science and Environmental Health Network, Ames, Iowa, USA; 17 Massachusetts General Hospital, Boston, Massachusetts, USA; 18 University of Miami Miller School of Medicine, Miami, Florida, USA; 19 New York State Department of Health, Albany, New York, USA; 20 National Institutes of Health, Bethesda, Maryland, USA; 21 Rollins School of Public Health, Atlanta, Georgia, USA; 22 Kaiser Permanente, Oakland, California, USA; 23 Harvard School of Public Health, Boston, Massachusetts, USA

**Keywords:** cholesterol, coffee, dairy products, diet, dopamine, fatty acids, metals, nonsteroidal anti-inflammatory drugs, Parkinson’s disease, pesticides, polychlorinated biphenyls, smoking, statins, urate

## Abstract

**Background:**

Parkinson’s disease (PD) is the second most common neurodegenerative disorder. People with PD, their families, scientists, health care providers, and the general public are increasingly interested in identifying environmental contributors to PD risk.

**Methods:**

In June 2007, a multidisciplinary group of experts gathered in Sunnyvale, California, USA, to assess what is known about the contribution of environmental factors to PD.

**Results:**

We describe the conclusions around which they came to consensus with respect to environmental contributors to PD risk. We conclude with a brief summary of research needs.

**Conclusions:**

PD is a complex disorder, and multiple different pathogenic pathways and mechanisms can ultimately lead to PD. Within the individual there are many determinants of PD risk, and within populations, the causes of PD are heterogeneous. Although rare recognized genetic mutations are sufficient to cause PD, these account for < 10% of PD in the U.S. population, and incomplete penetrance suggests that environmental factors may be involved. Indeed, interplay among environmental factors and genetic makeup likely influences the risk of developing PD. There is a need for further understanding of how risk factors interact, and studying PD is likely to increase understanding of other neurodegenerative disorders.

Parkinson’s disease (PD) is the second most common neurodegenerative disorder. The likelihood of developing PD increases with age. PD is rare before age 50. The average age of onset is in the mid- to late 60s (Bower et al. 1999; de Rijk et al. 1995; Marras and Tanner 2002; Van den Eeden et al. 2003). As the U.S. population ages, prevalence of this disabling disorder is expected to rise dramatically (Dorsey et al. 2007). Unfortunately, few valid data that address changes in PD incidence or prevalence over time are available. In fact, active case-finding efforts in communities detect as many as 10–40% of cases of PD for the first time, suggesting that underestimation of PD prevalence is common (de Pedro-Cuesta 1991; de Rijk et al. 1997).

The symptoms of PD are slowly progressive. Well-recognized clinical features of PD are slowed movements, tremor, rigidity, and difficulties with gait and balance. However, other features commonly occur, including changes in olfaction, autonomic function, cognitive function, affect, sleep, and energy level (Alves et al. 2005; Burn et al. 2006; Pfeiffer 1998; Stern et al. 1994).

In PD, specific neuronal populations degenerate. Neurodegeneration occurs in concert with the deposition of aggregates of the protein alpha synuclein in neuronal cell bodies and processes (Spillantini et al. 1998). The classical focus has been on dopamine-releasing cells in the substantia nigra, because dopamine replacement can partially correct some of the motor features of PD. It has long been known, however, that many other neuronal populations are also affected in PD. Recently, converging epidemiologic and pathologic data suggest that years or even decades before the onset of these classical features of PD, neurons outside of the central nervous system may be injured (Abbott et al. 2005, 2007; Braak et al. 2004; Langston 2006; Ross et al. 2008). If this is correct, current concepts of PD will need revision. Clarification may provide exciting new opportunities for treatment and intervention.

In the 1980s, the observation that intravenous exposure to 1-methyl-4-phenyl-1,2,3,6-tetrahydropyridine (MPTP) caused parkinsonism in humans offered important insights into environmental triggers (Langston et al. 1983). Even though relatively uncommon genetic mutations are sufficient to cause some cases of PD, twin studies conclude that the contribution of genetic makeup to the risk of most cases of PD is limited (Tanner et al. 1999; Wirdefeldt et al. 2004). Moreover, studies of uncommon genetic forms of PD have shown that changes in the structure of proteins that can lead to neural dysfunction, and death can also be caused independently by some environmental toxicants (Lee 2003; Purisai et al. 2005; Uversky et al. 2001; Vila et al. 2000). As a result, a general view has evolved that the vast majority of cases of PD are caused by environmental factors interacting with genetic makeup.

People with PD, their families, scientists, health care providers, and the general public are increasingly interested in identifying environmental contributors to PD with an eye toward not only more effective treatments but also prevention. What percentage of cases involves preventable causes? What precautionary interventions would be warranted and effective, and when must they be implemented? Can individuals destined to develop neurologic dysfunction due to PD be identified before typical motor symptoms manifest?

The current scientific literature does not provide conclusive answers to most of these and other relevant questions. But indications from epidemiology, basic neurobiology, and toxicology increasingly support the conclusion that a large portion of the risk of developing PD may be attributable to environmental exposures. Therefore, the risk of PD is theoretically reducible to the extent that some cases may be preventable.

## Purpose of Conference

Responding to these questions and concerns, a multidisciplinary group of experts gathered in Sunnyvale, California, USA, 26–28 June 2007, to assess what is known about the contribution of environmental factors to PD. Participants included toxicologists, epidemiologists, geneticists, neuroscientists, and medical practitioners. They were joined by representatives of PD advocacy groups and people with PD to review the state of environmental health science as it pertains to PD.

The purposes of the meeting were as follows:

- To review findings from diverse research disciplines concerning environmental factors that alone or in combination with genetic variables provide a biologic basis of PD
- To identify conclusions that could be drawn with confidence from existing data
- To identify plausible but uncertain conclusions
- To identify research gaps and needs and to describe features of a coherent research agenda.

Participants recognized the existence of various syndromes that may share some clinical and neurobiologic features with classic PD. Sometimes the term “parkinsonism” is used to refer to these syndromes. They often involve more extensive (or less specific) brain injury than is typically seen in classic PD and can be degenerative or nondegenerative (e.g., carbon monoxide–induced parkinsonism, carbon tetrachloride–induced parkinsonism, vascular parkinsonism). However, boundaries between PD and parkinsonism are evolving concepts. For this reason, participants were not asked to discuss and come to a consensus definition of PD.

Participants did not attempt to rank specific pathogenic mechanisms with respect to their relative importance in PD causation. Nonetheless, various combinations of alpha synuclein deposition, mitochondrial dysfunction, proteosome dysfunction, oxidative stress, and inflammation arose in discussions of potential contributors in causal pathways.

Participants were also not asked to address and did not consider *a*) an exhaustive list of toxicants that have been associated with PD (examples of toxicants not considered include organic solvents, electromagnetic fields); or *b*) factors that may influence the progression of PD (as differentiated from causes of onset of PD).

Over the course of the meeting, the following core points of consensus were identified, which we offer in this summary to acquaint scientists, medical professionals, public health advocates, and policy makers with the current state of understanding in the field, as seen by conference participants, and to help in identifying fruitful research strategies.

## Findings

### Sufficient evidence of a causal relationship

Based on existing evidence, we are confident of the following:

- PD is a complex disorder, and multiple different pathogenic pathways and mechanisms can ultimately lead to PD. Within the individual there are multiple determinants of PD risk, and within populations the causes of PD are heterogeneous. The interplay among environmental factors and genetic makeup likely influences the risk of developing PD (Chade et al. 2006; Warner and Schapira 2003).
- PD risk increases with age (Bower et al. 1999; de Rijk et al. 1995; Van den Eeden et al. 2003; Zhang et al. 2003).
- Studying PD is likely to increase understanding of other neurodegenerative disorders.
- Rare recognized genetic mutations are sufficient to cause PD (Singleton et al. 2003; Warner and Schapira 2003). However, collectively these genetic mutations account for < 10% of PD in the U.S. population. Moreover, even in these rare instances, incomplete penetrance suggests that environmental factors may be involved (Elbaz 2008).
- In addition to rare genetic mutations, PD or parkinsonism can also rarely be induced primarily by exposure to toxicants that directly target the area of the brain involved in PD. MPTP is an example of such a toxicant (Langston et al. 1983).

PD risk factors are categorized according to the terminology of the Institute of Medicine (IOM) for strength of evidence. IOM committees sometimes classify the evidence of association between exposure to a specific agent and a specific health outcome into five previously established categories, as set forth below. The group of experts gathered in Sunnyvale decided to use these categories as a means of describing their evaluation of the state of the evidence with respect to the influence of various factors on PD risk. Criteria for inclusion in each category are described at the outset.

Participants felt that these categories for describing the strength of evidence pertaining to individual risk factors were useful even though not based here on a systematic evidence-based literature review. Risk factors were assigned to the various categories based on consensus opinion of those attending the conference. Moreover, within each category there is no attempt to list the entries in any particular order.

### Sufficient evidence of an association

In this IOM category, evidence from available studies is sufficient to conclude that there is a positive association. A consistent positive association has been observed between exposure to a specific agent and a specific health outcome in human studies in which chance and bias, including confounding, could be ruled out with reasonable confidence. For example, several high-quality studies report consistent positive associations, and the studies are sufficiently free of bias, including adequate control for confounding.

- Men are at greater risk of PD than women (Bower et al. 1999; de Rijk et al. 1995; Van den Eeden et al. 2003). However, increased risk to a disease can occur because of inherently increased susceptibility, increased exposures to causal agent(s), or combinations of the two (Wooten et al. 2004). Current evidence is not sufficient to explain with confidence the increased risk in males.
- Evidence is sufficient to conclude with confidence that cigarette smokers have a lower risk of PD than nonsmokers (Hernan et al. 2002; Ritz et al. 2007). PD risk may also be lower for people who use other tobacco products, although the evidence is not as extensive or persuasive as for cigarette smokers, and conference participants were less confident in drawing conclusions.
- Evidence is also sufficient to conclude that male coffee drinkers have a lower risk of PD than females (Ascherio et al. 2004; Ross et al. 2000). For women coffee drinkers and people consuming other caffeinated beverages, evidence is limited, but a similar pattern seems to emerge (Ascherio et al. 2001).

Conference participants are confident of the associations but uncertain about the causal relationships or pathways by which smoking and coffee consumption might have a neuroprotective effect. Various biologic mechanisms have been proposed. For example, nicotine in cigarettes and caffeine in coffee are hypothesized to be agents that may confer a lower PD risk. However, noncausal explanations for these associations are also plausible (Hernan et al. 2002), uncertainties remain, and no consensus has been achieved. Nonetheless, further investigation into the biologic mechanisms by which female sex, cigarette smoking, and coffee consumption lower PD risk is warranted and may result in important insights into the etiology and progression of PD.

### Limited suggestive evidence of an association

In this IOM category, evidence from available studies suggests an association between exposure to a specific agent and a specific health outcome in human studies, but the body of evidence is limited by the inability to rule out chance and bias, including confounding, with confidence. For example, at least one high-quality study reports a positive association that is sufficiently free of bias, including adequate control for confounding. Other corroborating studies provide support for the association, but they were not sufficiently free of bias, including confounding. Alternatively, several studies of less quality show consistent positive associations, and the results are probably not due to bias, including confounding.

Available scientific studies suggest an association between a number of different factors and PD risk. Here, the data are limited but tend to point toward a valid association with PD risk. Causal mechanisms explaining the associations, if they exist, are not well understood.

- People with higher levels of physical activity have a reduced risk of PD (Thacker et al. 2008).
- Men, and possibly women, with higher blood urate levels have a reduced risk of PD (Davis et al. 1996; Weisskopf et al. 2007).
- People taking nonsteroidal anti-inflammatory drugs have a lower risk of PD (Powers et al. 2008a; Wahner et al. 2007).
- Men with high dietary intake of dairy products have an increased risk of PD (Chen et al. 2002; Park et al. 2005).
- Farmers and agricultural workers have an increased risk of PD (Hertzman et al. 1994; Gorell et al. 1998; Tuchsen and Jensen 2000). Epidemiologic studies often classify study participants according to their occupation and examine for associations between occupations and outcomes of interest. In these cases, inferences about potential exposures that characterize those occupations may be drawn, but they remain as inferences and should not be confused with estimates of exposure to specific environmental agents.
- People exposed to pesticides have an increased risk of PD (Dick et al. 2007; Gorell et al. 1998; Hancock et al. 2008; Kamel et al. 2007). However, epidemiologic study designs may classify study participants according to reported exposures to classes of chemicals or other agents. In these cases, associations between the class of environmental agents and outcome of interest may be identified, but in most cases specific agents that account for the association cannot be further identified from those data. Meeting participants note the evidence suggesting a direct association between pesticide exposure and PD risk but are unable to draw any conclusions about specific agents that may be responsible.
- People with traumatic brain injury have an increased risk of PD (Bower et al. 2003, Dick et al. 2007; Goldman et al. 2006).
- Certain variants of genes can modify the risk of PD. The risk may be higher or lower depending on the variant (Warner and Schapira 2003).

### Inadequate/insufficient evidence to determine whether an association exists

In this IOM category, evidence from available studies is of insufficient quantity, quality, or consistency to permit a conclusion regarding the existence of an association between exposure to a specific agent and a specific health outcome in humans.

- People with higher dietary intake of poly-unsaturated fatty acids have lower risk of PD (de Lau et al. 2005).
- People with higher blood cholesterol have lower risk of PD (Hu et al. 2008; Simon et al. 2007).
- Dietary sources of urate lower the risk of PD (Annanmaki et al. 2007; Gao et al. 2008).
- People taking statins are at lower risk of PD (Becker et al. 2008; Wahner et al. 2008).
- Postmenopausal women taking exogenous estrogen are at reduced risk of PD (Currie et al. 2004; Popat et al. 2005).
- People with increased body mass index or body fat are at increased risk of PD (Hu et al. 2006; Logroscino et al. 2007).
- Women occupationally exposed to poly-chlorinated biphenyls (electrical capacitance workers) are at increased risk of PD (Prince et al. 2006; Steenland et al 2006).
- People with higher educational level are at higher risk of PD (Frigerio et al. 2005).
- People exposed to some specific pesticides have an increased risk of PD (paraquat, maneb, dieldrin) (Dick 2006; Drechsel and Patel 2008; Kamel et al. 2007). *In vitro* and *in vivo* laboratory animal studies of some specific pesticides (e.g., rotenone, paraquat, maneb) demonstrate toxicity to nigral dopaminergic neurons and reveal biologic mechanisms by which exposure to those pesticides is plausibly linked to PD risk (Thiruchelvam et al. 2000; Uversky 2004).
- People exposed to some heavy metals have an increased risk of PD (Gorell et al. 1998; Mergler et al. 1994).

### Limited suggestive evidence of no association

In this IOM category, evidence from available studies is consistent in not showing a positive association between exposure to a specific agent and a specific health outcome after exposure of any magnitude. A conclusion of no association is inevitably limited to the conditions, magnitudes of exposure, and length of observation in the available studies. The possibility of a very small increase in risk after exposure studied cannot be excluded.

Welding is not associated with risk of PD (Marsh and Gula 2006; Santamaria et al. 2007).

### Consensus not reached on category of association

In this IOM category, if the entire committee did not agree on a conclusion, then the association was not assigned a category.

- People living in rural areas have/do not have (Kuopio et al. 1999; Priyadarshi et al. 2001) an increased risk of PD.
- Vitamin E, vitamin C, and carotenoids are/are not associated with PD risk (Etminan et al. 2005; Zhang et al. 2002).
- Dietary saturated fats are/are not associated with risk of PD (de Lau et al. 2005; Gao et al. 2007; Powers et al. 2008b).
- Well water drinking is/is not associated with PD risk (Kuopio et al. 1999; Priyadarshi et al. 2001).
- Race/ethnicity is/is not associated with PD risk (Mayeux et al 1995; Van den Eeden et al. 2003; Zhang and Román 1993).

## Research Needs

- Well-designed case -control studies with sufficient power are needed—perhaps through consortia or multicenter—as well as strict attention to bias and confounding.
- Consider the question of appropriate control groups. Consider alternatives to traditional sources of controls used in the past such as random digit dialing and centers for Medicare and Medicaid services, as these sources are now often impractical because of privacy and other considerations.
- PD registries, particularly inclusive, legally mandated, population-based registries, are essential to description of demographic, geographic, and temporal patterns and trends.
- Diagnostic criteria for cases need to be well defined in studies. Criteria and disease definition may vary depending on the specific hypothesis being tested. Similarly, characterization of clinical features is critical. Some risk factors may be specific for classic PD, whereas others may be common for all types of parkinsonism.
- Biomarkers of disease are needed, beginning with early stages.
- More consistent attempts are needed to look at both susceptibility genes and environmental agents in combination.
- Better measures of the exposures of interest are needed.

## Research Questions

In addition to known risk factors for PD, conference participants identified a number of others in need of further investigation. Even for those that are well established, however, there is a need for additional information about mechanisms by which they influence PD risk. Moreover, in addition to individual risk factors, conference participants recognized a need for further understanding of how risk factors interact as they contribute to the multiple pathogenic pathways that may ultimately result in PD.
